# Insulin resistance in women with recurrent miscarriage: a systematic review and meta-analysis

**DOI:** 10.1186/s12884-022-05256-z

**Published:** 2022-12-08

**Authors:** Wang-Yu Cai, Xi Luo, Hou-Yi Lv, Kai-You Fu, Jian Xu

**Affiliations:** 1 Fourth Affiliated Hospital, Zhejiang University, School of Medicine, No. 1 Shang Cheng Avenue, Yiwu, 322000 Zhejiang China; 2 Department of Obstetrics and Gynecology, The Second Affiliated Hospital of Zhejiang Chinese Medical University, Hangzhou, China; 3 International Institutes of Medicine, The Fourth Affiliated Hospital of Zhejiang University School of Medicine, Yiwu, Zhejiang China; 4 The First Affiliated Hospital, Zhejiang University, School of Medicine, Hangzhou, Zhejiang China; 5 Women’s Hospital, Zhejiang University, School of Medicine, Hangzhou, Zhejiang China

**Keywords:** Recurrent pregnancy loss, Insulin resistance, HOMA-IR, GI ratio, Systematic review

## Abstract

**Purpose:**

This review aimed to investigate the association of insulin resistance (IR) in women with recurrent pregnancy loss compared to women with normal pregnancy history.

**Methods:**

PubMed, EMBASE, the Web of Science and Google Scholar databases were accessed to collect published observational studies that compared IR of recurrent pregnancy loss women with healthy women until the 6^th^ of October 2022. Outcomes assessed in this review and meta-analysis included fasting blood glucose, fasting plasma insulin, homeostasis model assessment for IR, glucose to insulin ratio. Mean differences, odds ratios with 95% confidence interval were pooled using the fixed or random effect models. Sensitivity analyses were performed to validate the robustness of the results. Review Manager version 5.4.1 and Stata version 8.0 were used.

**Results:**

A total of nineteen studies involving 4453 individuals were included. Recurrent pregnancy loss patients presented significantly higher fasting blood glucose, fasting plasma insulin, homeostasis model assessment for IR, and lower glucose to insulin ratios. Additionally, recurrent pregnancy loss patients had higher rates of IR as defined by abnormal fasting plasma insulin, homeostasis model assessment for IR, and glucose to insulin ratio. Sensitivity analyses validated the robustness of the results.

**Conclusion:**

In the current review, we show that recurrent pregnancy loss is associated with a higher degree of IR and highlight the importance of screening and treatment of IR.

**Supplementary Information:**

The online version contains supplementary material available at 10.1186/s12884-022-05256-z.

## Background

Recurrent pregnancy loss (RPL) affects approximately 2–5% of women [1]. Common causes of RPL include chromosomal abnormalities [2], uterine abnormalities [3], endocrine imbalances [4], autoimmune factors such as antiphospholipid syndrome [5], thrombophilia [6], and environmental factors such as tobacco use, caffeine intake, and alcohol use [7–9]. However, despite comprehensive evaluation, more than 50% of cases remain unexplained [10]. American Heart Association recommend to incorporate obstetric complications including preeclampsia, gestational diabetes, or pregnancy-induced hypertension as risk factors for development of cardiovascular disease in women [11]. A history of recurrent miscarriages was associated with higher risk of non-fatal and fatal stroke in a recent review [12]. These evidences suggest that cardiovascular and metabolic abnormalities may also predispose to RPL.

Previous studies have shown that insulin resistance (IR) may play a role in female reproduction. Tian et al*.* suggested that IR was an independent risk factor for spontaneous abortion in women who received infertility treatment [13]. Additionally, several studies show that IR may play a role in polycystic ovary syndrome (PCOS) since their underlying connection [14, 15]. For example, a recent systematic review and meta-analysis of the literature highlighted IR as a risk factor for spontaneous abortion in PCOS patients undergoing assisted reproduction [16]. Hyperinsulinemia and IR are also associated with poor reproductive outcomes in PCOS patients undergoing ovulation induction [17]. Previous studies have shown that insulin functioning and metabolism are changed during pregnancy and that IR has serious implications for pregnancy outcomes and long-term morbidity for both the mother and fetus [18]. However, the potential effect of IR on RPL remains to be elucidated. Thus far, several case–control studies have reported differences in IR between women with RPL and healthy controls. However, no comprehensive review exists on this topic.

In the current review, we aim to run a meta-analysis and systematically review relevant literature to establish the role of IR status in RPL patients relative to healthy controls.

## Methods and materials

### Literature search strategy

This systematic review and meta-analysis was constructed according to the Preferred Reporting Items for Systematic Reviews and Meta-analyses (PRISMA) [19] (Supplementary Table 1). The protocol was previously registered (INPLASY2021110055). Major electronic databases including PubMed, Embase, and Web of Science were used to source relevant literature published until the 6^th^ of October 2022. Key search terms included: “recurrent miscarriage”, “insulin resistance”, and “case–control” (Supplementary Table 2). Google Scholar was also searched for related articles that may not be in the search databases. References from all included studies were also assessed to identify relevant articles not captured by the electronic searches.

### Inclusion and exclusion criteria

Observational studies that compared IR parameters in RPL patients to control women with normal pregnancy history were included. IR parameters included fasting blood glucose (FBG), fasting insulin (FIN), homeostasis model assessment for insulin resistance (HOMA-IR), glucose to insulin ratio (GI ratio). Only studies that were published in English were included. Review articles, opinions, book chapters, letters, published abstracts, animal studies, case reports were excluded. For studies with no suitable control women (e.g. healthy pregnant women, women with healthy pregnancy history) to RPL women, they were excluded.

### Study selection

Two authors (WYC and XL) independently scrutinized the titles and abstracts of all potential studies to strictly identify relevant studies according to the inclusion and exclusion criteria. Relevant studies that were considered for inclusion were then carefully reviewed**.** Any disagreement between the two authors was resolved by a third author (JX).

### Data extraction

Two authors (WYC and XL) independently extracted data using the following format: the first author, year of publication, geographic region, sample size, study design, age of case and control, outcome measures, exclusion of known factors which contribute to RPL, and matched factors were recorded. Where a study with two or more publications was identified, only the most comprehensive or the most recent version was included. For publications that reported median and interquartile range, the mean and standard deviation was estimated [20].

### Quality assessment

The quality of eligible observational studies was assessed using the Newcastle–Ottawa scale (NOS) [21]. The NOS assesses studies by scoring three aspects: viz selection, comparability, and exposure. The total NOS is scored out of 9 (the higher the score, the better). Each article was awarded a score out of four for selection bias (adequate definition of case, representativeness of the case, selection of control, definition of control), two for comparability (comparability between case and control), and four for bias in the exposure (ascertainment of exposure, consistency of the method of ascertainment for case and control, and non-response rate).

### Statistical analyses

Review Manager version 5.4.1 and Stata version 8.0 were used to analyze the extracted data. The mean difference (MD) or odds ratio (OR) with 95% confidence interval (CI) were pooled to measure the effect size. The heterogeneity of studies was measured using the I^2^ index: a value below 40% indicated no heterogeneity; a value greater than 40% indicated the occurrence of heterogeneity. When no heterogeneity was observed, the fixed-effects model was used. The random-effects model was used when heterogeneity existed. Publication bias was assessed using funnel plot asymmetry, Egger’s (number of included studies <  = 10) or Begg’s (number of included studies > 10) line regression test. To measure the effect of confounders on the effect size, subgroup analysis was performed. To confirm the robustness of the results, a sensitivity analysis was performed by systematically excluding each study included in the analysis. A P-value less than 0.05 was considered statistically significant.

## Results

**Fig. 1:**
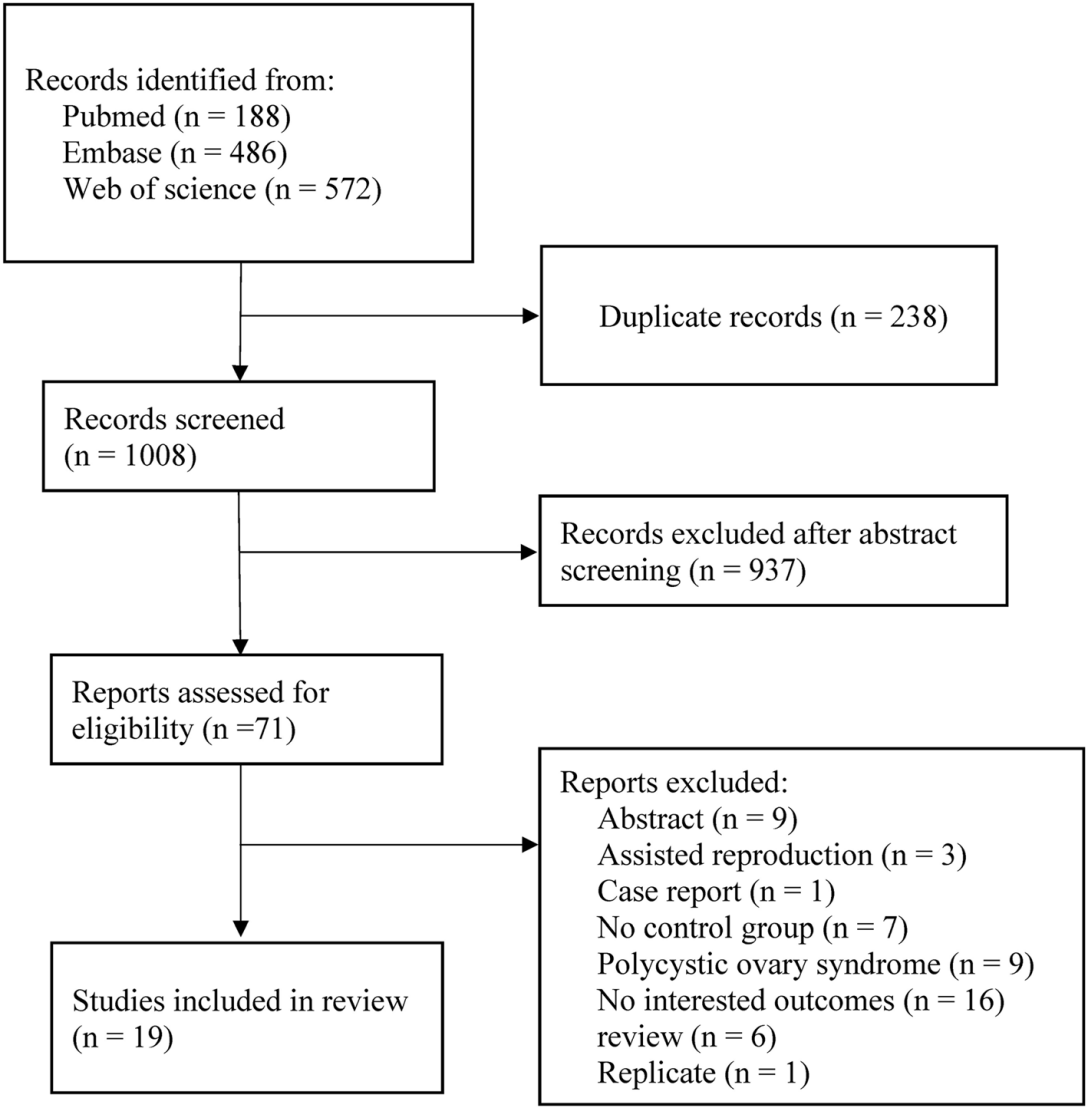
Flowchart for selecting studies

Following title and abstract screening of the literature search results, 1246 total studies were assessed of which 238 were duplicates and 937 were considered irrelevant. Of the remaining 71 records, 52 records were excluded due to only abstract (*n* = 9), assisted reproduction (*n* = 3), case report (*n* = 1), no control group (*n* = 7), PCOS (*n* = 9), no interested outcomes (*n* = 16), review (*n* = 6), replicate (*n* = 1) (Fig. 1). No additional studies were identified through Google Scholar and article references. Therefore, a total of 19 studies were eligible for data extraction and were included in the present meta-analysis [22–40] (Fig. 1).

### Characteristics of included studies

The studies included in this review were published between 2002 and 2022, and consist of 2223 RPL women and 2230 control women (Table 1). Among the studies assessed, six were conducted in the Middle East, six in Asia, three in Africa, two in North America, and two in Europe. All studies were case-controlled. The participant's mean age of RPL cases ranged from 24.8 to 35.8 years. Eight studies defined RPL as three or more consecutive miscarriages, eleven studies defined RPL as two or more consecutive miscarriages. Fourteen studies excluded known factors for RPL while five studies did not exclude such factors. Four studies measured outcomes during pregnancy, while fifteen studies measured outcomes in women who were not pregnant. Ten studies matched for one or more confounders, while nine studies did not match for confounding factors at all. FBG was assessed in eighteen studies. FIN was assessed in sixteen studies. HOMA-IR was assessed in eight studies. The GI ratio was assessed in five studies. The qualitative assessment of the data revealed that the data of all included studies were of medium to high quality (Table 2).

**Table 1:** Characteristics of included studies

**Author/year published**	**Country**	**RPL**		**Control**		**Definition of RM**	**Outcome measurements**	**Measurement timing**	**Exclusion of known cause**	**Matched variables**
		**Number of cases**	**Age of cases**	**Number of controls**	**Age**					
Craig 2002 [26]	United States	74	32.7 ± 5.4	74	32.8 ± 6.0	> = 2 times before 20 weeks or fetal weight of less than 500 g	FBG, FIN, GI ratio, IR status	Not pregnant	No	Age, weight, race
Diejomaoh 2007 [27]	Kuwait	35	28.6 ± 4.3	30	27.5 ± 4.6	> = 3 times	FBG, FIN, HOMA-IR, GI ratio, IR status	Not pregnant	No	Age, ethnic, BMI
Baban 2010 [23]	Iraq	64	27.53 ± 8.84	51	28.72 ± 8.18	> = 3 times	FBG, FIN	pregnant	Yes	None
Celik 2011 [25]	Turkey	64	29.5 ± 6.0	64	30.7 ± 6.0	> = 2 times before 20 weeks	FBG, FIN, HOMA-IR, GI ratio, IR status	Not pregnant	Yes	Age, BMI
Wang 2011 [38]	China	97	30.81 ± 4.01	52	29.15 ± 4.62	> = 2 times	FBG, FIN, HOMA-IR	Gestational age of 5–13 weeks	Yes	None
Maryam 2012 [36]	Iran	50	28.15 ± 4.76	50	28.28 ± 4.77	> = 3 times before 24 weeks	FBG, FIN, GI ratio, IR status	Not pregnant	No	Age, BMI
Hong 2013 [30]	China	161	27.1 ± 2.9	465	26.8 ± 3.0	> = 2 times	FBG, FIN, HOMA-IR, GI ratio	first trimester pregnancy	No	None
Ispasoiu 2013 [31]	Romania	65	30.12 ± 4.904	53	29.36 ± 5.274	> = 2 times	FBG, FIN	Not pregnant	Yes	None
Kazerooni 2013 [34]	Iran	60	24.8 ± 3.9	60	24.6 ± 4.7	> = 3 times before 20 weeks	FBG, FIN	10th day of the menstrual cycle	Yes	Age, BMI, parity
Ota 2014 [37]	United States	63	35.8 ± 4.8	30	35.1 ± 4.1	> = 3 times before 20 weeks	FBG, FIN	Periovulatory phase of menstrual cycle	Yes	Age
El-Dorf 2016 [29]	Egypt	35	27.08 ± 4.102	35	28.12 ± 4.825	> = 2 times at first trimester	FIN	pregnancy between 5 and 12 weeks	Yes	None
Jiao 2016 [33]	China	154 152	35.2 ± 3.7 35.6 ± 4.1	155 151	35.1 ± 4.5 35.7 ± 3.8	> = 3 times before 20 weeks	FBG	Not pregnant	Yes	Age
Azizi 2019 [22]	Iran	28	27.39 ± 4.12	42	26.31 ± 3.81	> = 3 times before 20 weeks	FBG	luteal phase of menstrual cycle	Yes	None
Lin 2019 [35]	China	403	29.58 ± 5.48	342	29.88 ± 5.28	> = 2 times	FBG, FIN, HOMA-IR	Not pregnant	Yes	None
Bahia 2020 [24]	Tunisia	332	35.8 ± 7.7	286	32.5 ± 6.2	> = 3 times before 20 weeks	FBG	normo-ovulatory cycle	Yes	Age, menopause status, ethnic
Edugbe 2020 [28]	Nigeria	80	28.09 ± 6.14	80	28.10 ± 6.21	> = 2 times before 13 weeks	FBG, FIN, HOMA-IR	Not pregnant	Yes	Age
Jiang 2021 [32]	China	133	34.1 ± 3.9	140	33.4 ± 2.9	> = 2 times at first trimester	FBG, FIN, HOMA-IR	at least 12 weeks after the last abortion	Yes	None
Habets 2022 [39]	Netherlands	65 58	32.6 ± 4.5 33.5 ± 4.6	20	33.1 ± 6.8	> = 2 times before 24 weeks	FBG, FIN	at least 3 months after miscarriage	None	None
Singh 2022 [40]	India	50	25.1 ± 3.0	50	25.8 ± 2.8	> = 2 times	FBG, FIN, HOMA-IR, IR status	Not pregnant	Yes	Age, BMI

The data are presented as mean ± standard deviation, median (quartile1, quartile 3) and median [range]

*RM* recurrent pregnancy loss, *BMI* body mass index, *FBG* fasting blood glucose, *FIN* fasting insulin, *HOMA-IR* homeostatic model assessment for insulin resistance, *GI ratio* glucose to insulin ratio, *IR* insulin resistance

**Table 2:** Quality of included studies

	**Selection**				**Comparability**		**Exposure**			
**Author/year published**	**Adequate Case Definition**	**Representative of cases**	**Selection of controls**	**Definition of Controls**	**Age**	**Other factors**	**Ascertainment of exposure**	**Same ascertainment**	**Non-Response Rate**	**Total**
Craig 2002 [26]	*	*		*	*	*	*	*	*	8
Diejomaoh 2007 [27]	*	*		*	*	*	*	*	*	8
Baban 2010 [23]	*			*			*	*	*	5
Celik 2011 [25]	*	*		*	*	*	*	*	*	8
Wang 2011 [38]	*			*			*	*	*	5
Maryam 2012 [36]	*			*	*	*	*	*	*	7
Hong 2013 [30]	*			*			*	*	*	5
Ispasoiu 2013 [31]	*			*			*	*	*	5
Kazerooni 2013 [34]	*				*	*	*	*	*	6
Ota 2014 [37]	*			*	*		*	*	*	6
El-Dorf 2016 [29]	*			*			*	*	*	5
Jiao 2016 [33]	*			*	*		*	*	*	6
Azizi 2019 [22]	*						*	*	*	4
Lin 2019 [35]	*			*			*	*	*	5
Bahia 2020 [24]	*		*	*	*	*	*	*	*	8
Edugbe 2020 [28]	*			*	*		*	*	*	6
Jiang 2021 [32]	*			*			*	*	*	5
Habets 2022 [39]	*			*			*	*	*	5
Singh 2022 [40]	*			*	*	*	*	*		6

#### FBG

**Fig. 2:**
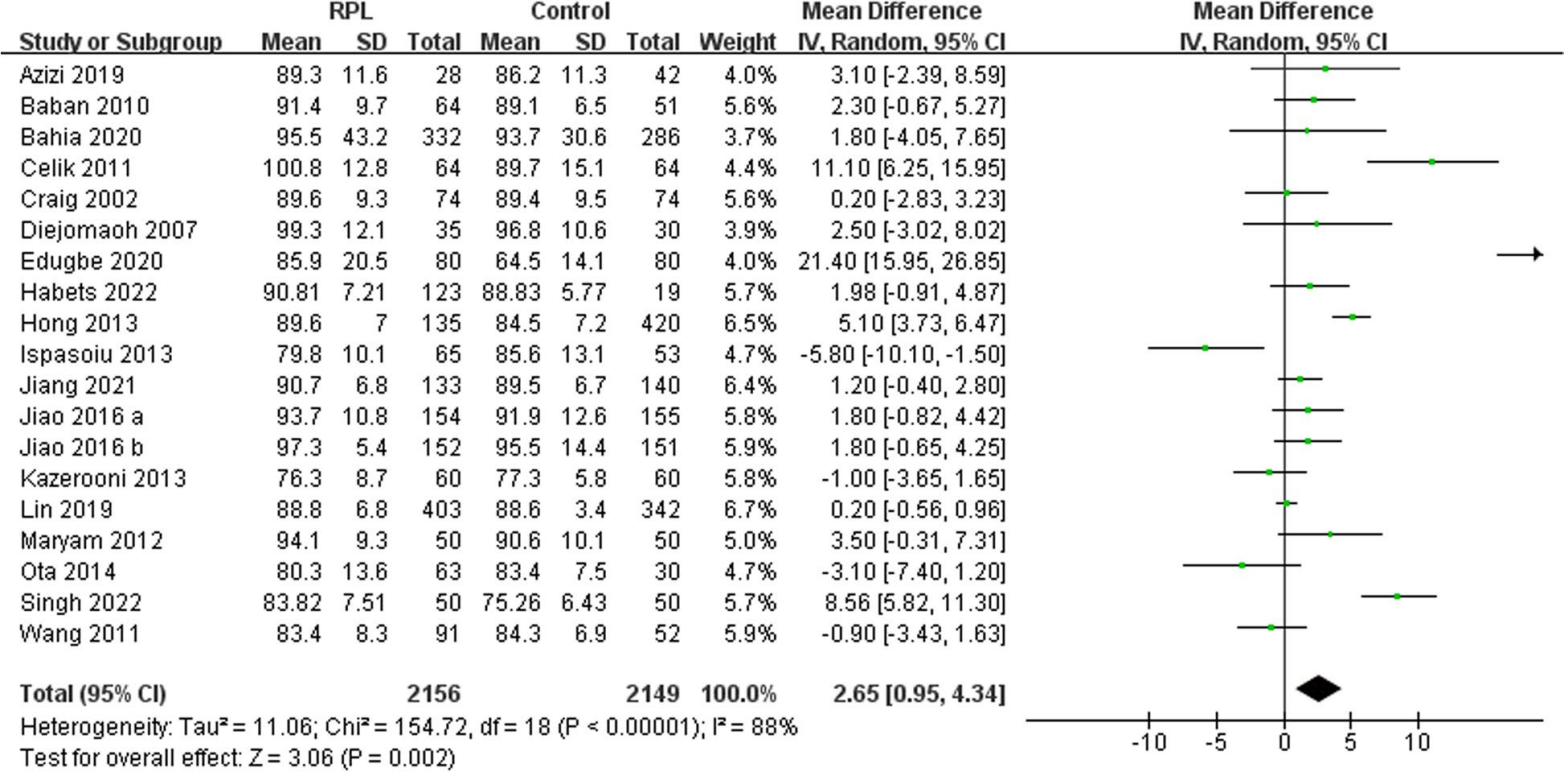
Meta-analysis of FBG between the recurrent pregnancy loss and control groups

FBG was measured in eighteen of the studies (Fig. 2). Nineteen comparisons were made as one study had two comparisons. Our meta-analysis indicated higher levels of FBG among RPL women compared to the control group (I^2^ = 88% [83%-92%]). The funnel plot showed no obvious asymmetry indicative of a lack of evidence of publication bias (Supplementary Fig. 1). Furthermore, the Begg’s line regression test did not indicate publication bias (z = 0.91, *P* = 0.363). Additionally, the sensitivity analysis did not identify any single study which significantly altered the effect size. Subgroup analyses indicated that the geographic region, the definition of RPL, whether the patients were matched for confounders, measurement timing, and the exclusion of known factors were not associated with between-study heterogeneity (Table 3).

**Table 3:** Subgroup analyses for the association between FBG and RPL

					**Test for subgroup differences**		
**Subgroup**	**No. of comparisons**	**MD (95%CI)**	**I**^**2**^** (%)**	***P*-value for heterogeneity**	**Chi**^**2**^	***P***	**I**^**2**^** (%)**
Region							
Asia	7	2.48 [0.33, 4.63]	91	< 0.00001	6.31	0.18	36.6
Africa	2	11.63 [-7.58, 30.84]	96	< 0.00001			
Europe	2	-1.74 [-9.36, 5.88]	88	0.003			
America	2	-1.08 [-4.24, 2.07]	34	0.22			
Middle East	6	3.49 [-0.15, 7.13]	74	0.002			
Definition of RPL							
> = 2	10	3.82 [1.17, 6.47]	94	< 0.00001	2.89	0.09	65.4
> = 3	9	1.27 [0.00, 2.54]	16	0.30			
Matched confounders							
No	8	1.09 [-0.84, 3.02]	87	< 0.00001	2.64	0.10	62.2
Yes	11	4.20 [0.98, 7.41]	89	< 0.00001			
Measurement timing							
During pregnancy	3	2.12 [-2.50, 6.73]	88	0.0002	0.06	0.80	0
Not during pregnancy	16	2.75 [0.80, 4.70]	88	< 0.00001			
Exclusion of known factors							
Yes	14	2.66 [0.54, 4.77]	9	< 0.00001	0.02	0.89	0
No	5	2.88 [0.72, 5.03]	62	0.03			

#### FIN

**Fig. 3:**
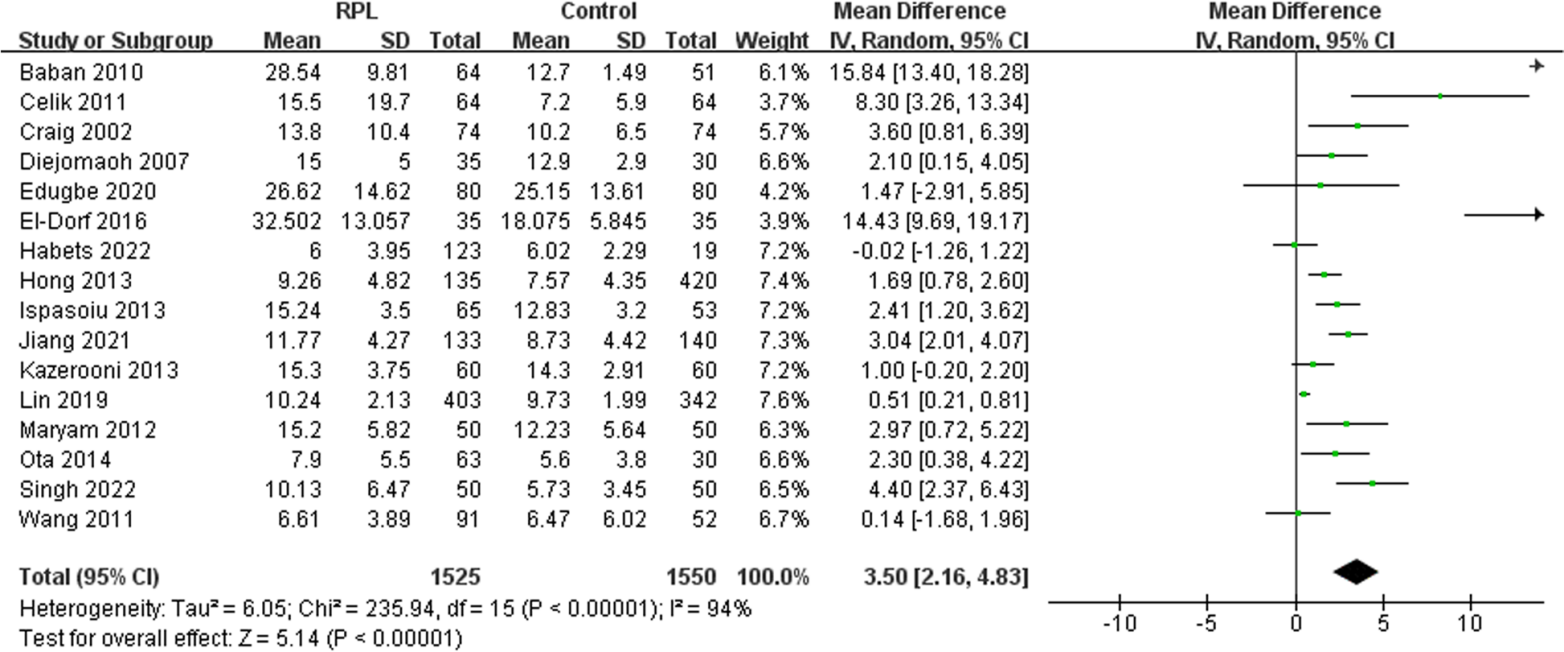
Meta-analysis of FIN between the recurrent pregnancy loss and control groups

FIN was measured in sixteen studies included in this review (Fig. 3). Our meta-analysis showed higher levels of FIN among RPL women compared to the control group (I^2^ = 94% [92%-96%]). The funnel plots showed possible asymmetry (Supplementary Fig. 2); however, the Begg’s line regression test did not indicate publication bias (z = 1.53, *P* = 0.125). Additionally, the sensitivity analysis did not identify any single study which altered the effect size. Subgroup analyses indicated that factors such as whether the patients were matched for confounders and the exclusion of known factors associated with RPL were correlated with between-study heterogeneity (*P* = 0.0009; *P* = 0.02) (Table 4).

**Table 4:** Subgroup analyses for the association between FIN and RPL

					**Test for subgroup differences**		
**Subgroup**	**No. of comparisons**	**MD (95%CI)**	**I**^**2**^** (%)**	***P*-value for heterogeneity**	**Chi**^**2**^	***P***	**I**^**2**^** (%)**
Region							
Asia	5	1.86 [0.52, 3.19]	89	< 0.00001	4.08	0.40	2
Africa	2	7.92 [-4.78, 20.61]	94	< 0.0001			
Europe	2	1.20 [-1.18, 3.58]	87	0.006			
America	2	2.72 [1.13, 4.30]	0	0.45			
Middle East	5	5.93 [0.71, 11.16]	97	< 0.00001			
Definition of RPL							
> = 2	11	2.63 [1.43, 3.83]	89	< 0.00001	0.81	0.37	0
> = 3	5	4.78 [0.25, 9.31]	97	< 0.00001			
Matched confounders							
No	8	1.02 [0.76, 1.27]	97	< 0.00001	11.00	0.0009	90.9
Yes	8	2.33 [1.60, 3.05]	55	0.03			
Measurement timing							
During pregnancy	4	7.82 [1.00, 14.63]	98	< 0.00001	2.55	0.11	60.8
Not during pregnancy	12	2.21 [1.25, 3.16]	82	< 0.00001			
Exclusion of known factors							
Yes	11	4.42 [2.45, 6.52]	96	< 0.00001	5.25	0.02	81.0
No	5	1.75 [0.58, 2.91]	60	0.04			

#### HOMA-IR

**Fig. 4:**
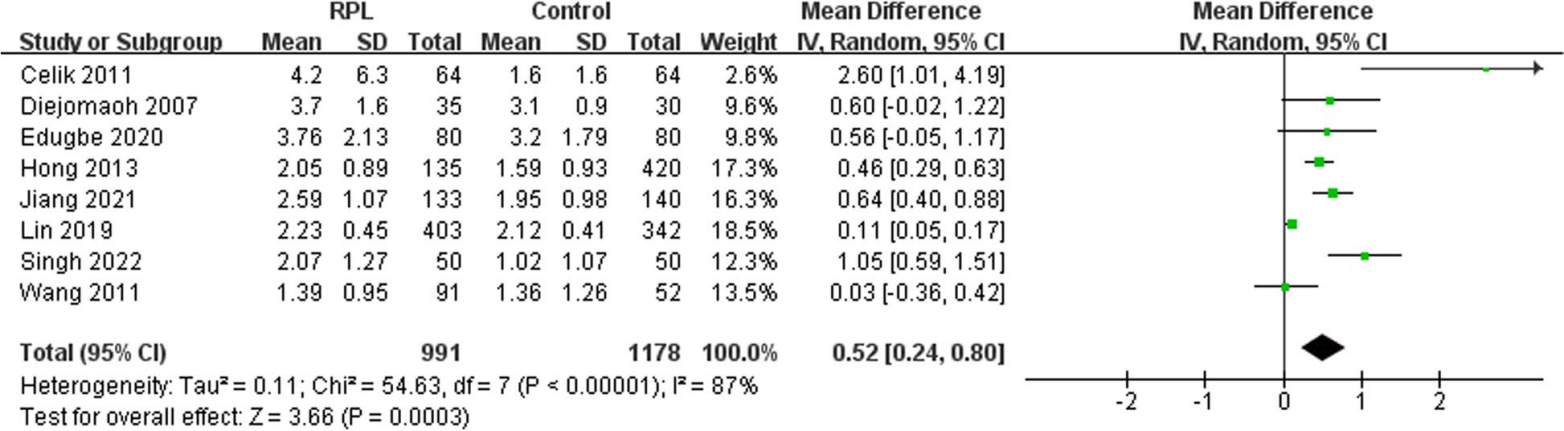
Meta-analysis of HOMA-IR between the recurrent pregnancy loss and control groups

The meta-analysis of eight studies revealed a significantly higher level of HOMA-IR in RPL patients compared to healthy controls (I^2^ = 85% [72%-92%]) (Fig. 4). The funnel plots showed no obvious asymmetry indicative of a lack of publication bias (Supplementary Fig. 3). The Egger’s line regression test did not indicate publication bias for HOMA-IR (t = 2.50, *P* = 0.054). Furthermore, the sensitivity analysis did not identify any single study which altered the effect size. Subgroup analyses indicated that the geographic region, the definition of RPL, whether the patients were matched for confounders, measurement timing, and the exclusion of known factors were not associated with between-study heterogeneity (Table 5).

**Table 5:** Subgroup analyses for the association between HOMA-IR and RPL

					**Test for subgroup differences**		
**Subgroup**	**No. of comparisons**	**MD (95%CI)**	**I**^**2**^** (%)**	***P*-value for heterogeneity**	**Chi**^**2**^	***P***	**I**^**2**^** (%)**
Region							
Asia	5	0.43 [0.13, 0.73]	91	< 0.00001	1.15	0.56	0
Africa	1	0.56 [-0.05, 1.17]					
Middle East	2	1.46 [-0.48, 3.40]	81	0.02			
Definition of RPL							
> = 2	7	0.51 [0.22, 0.81]	89	< 0.00001	0.06	0.80	0
> = 3	1	0.60 [-0.02, 1.22]					
Matched confounders							
No	4	0.32 [0.04, 0.60]	90	< 0.00001	3.96	0.05	74.8
Yes	4	0.92 [0.40, 1.43]	56	0.08			
Measurement timing							
During pregnancy	2	0.28 [-0.13, 0.70]	74	0.05	1.72	0.19	41.8
Not during pregnancy	6	0.68 [0.26, 1.10]	89	< 0.00001			
Exclusion of known factors							
Yes	6	0.42 [0.21, 0.64]	72	0.003	0.24	0.62	0
No	2	0.49 [0.31, 0.67]	0	0.85			

### GI ratio

**Fig. 5:**
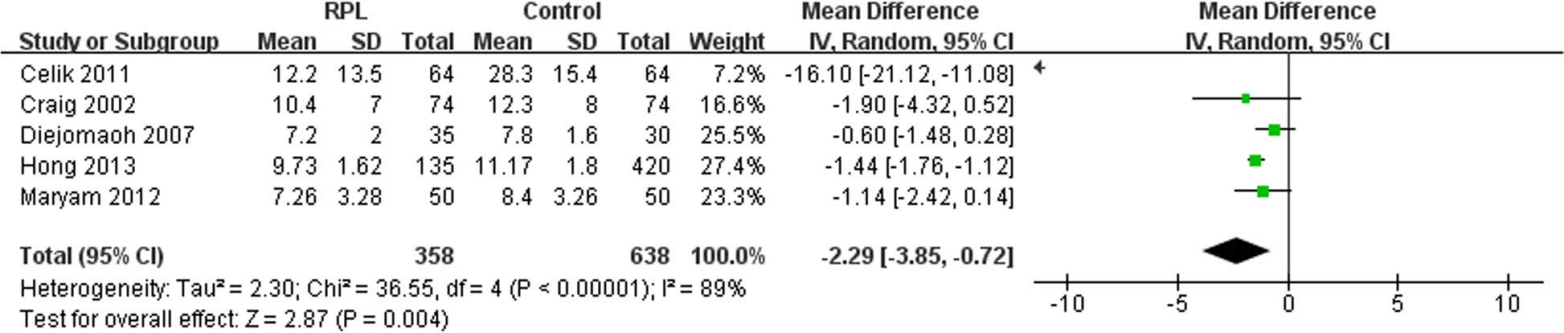
Meta-analysis of GI ratio between the recurrent pregnancy loss and control groups

The meta-analysis of five studies revealed a significantly lower GI ratio in RPL patients compared to the controls (I^2^ = 89% [77%-95%]) (Fig. 5). The funnel plots showed no obvious asymmetry indicative of a lack of publication bias (Supplementary Fig. 4). The Egger’s line regression test did not indicate publication bias (t = -0.93, *P* = 0.420). Additionally, the sensitivity analysis did not identify any single study which altered the effect size. Subgroup analyses indicated that the exclusion of known factors associated with RPL was associated with between-study heterogeneity (*P* < 0.00001) (Table 6).

**Table 6:** Subgroup analyses for the association between GI ratio and RPL

					**Test for subgroup differences**		
**Subgroup**	**No. of comparisons**	**MD (95%CI)**	**I**^**2**^** (%)**	***P*-value for heterogeneity**	**Chi**^**2**^	***P***	**I**^**2**^** (%)**
Region							
Asia	1	-1.44 [-1.76, -1.12]			2.48	0.29	19.2
America	1	-1.90 [-4.32, 0.52]					
Middle East	3	-4.47 [-8.32, -0.61]	94	< 0.00001			
Definition of RPL							
> = 2	3	-5.70 [-10.98, -0.42]	94	< 0.00001	3.28	0.07	69.5
> = 3	2	-0.77 [-1.49, -0.05]	0	0.50			
Matched confounders							
No	1	-1.44 [-1.76, -1.12]			2.02	0.16	50.6
Yes	4	-3.61 [-6.59, -0.64]	92	< 0.00001			
Measurement timing							
During pregnancy	1	-1.44 [-1.76, -1.12]			2.02	0.16	50.6
Not during pregnancy	4	-3.61 [-6.59, -0.64]	92	< 0.00001			
Exclusion of known factors							
Yes	1	-16.10 [-21.12, -11.08]			33.14	< 0.00001	97.0
No	4	-1.34 [-1.63, -1.04]	12	0.33			

### IR status

**Fig. 6:**
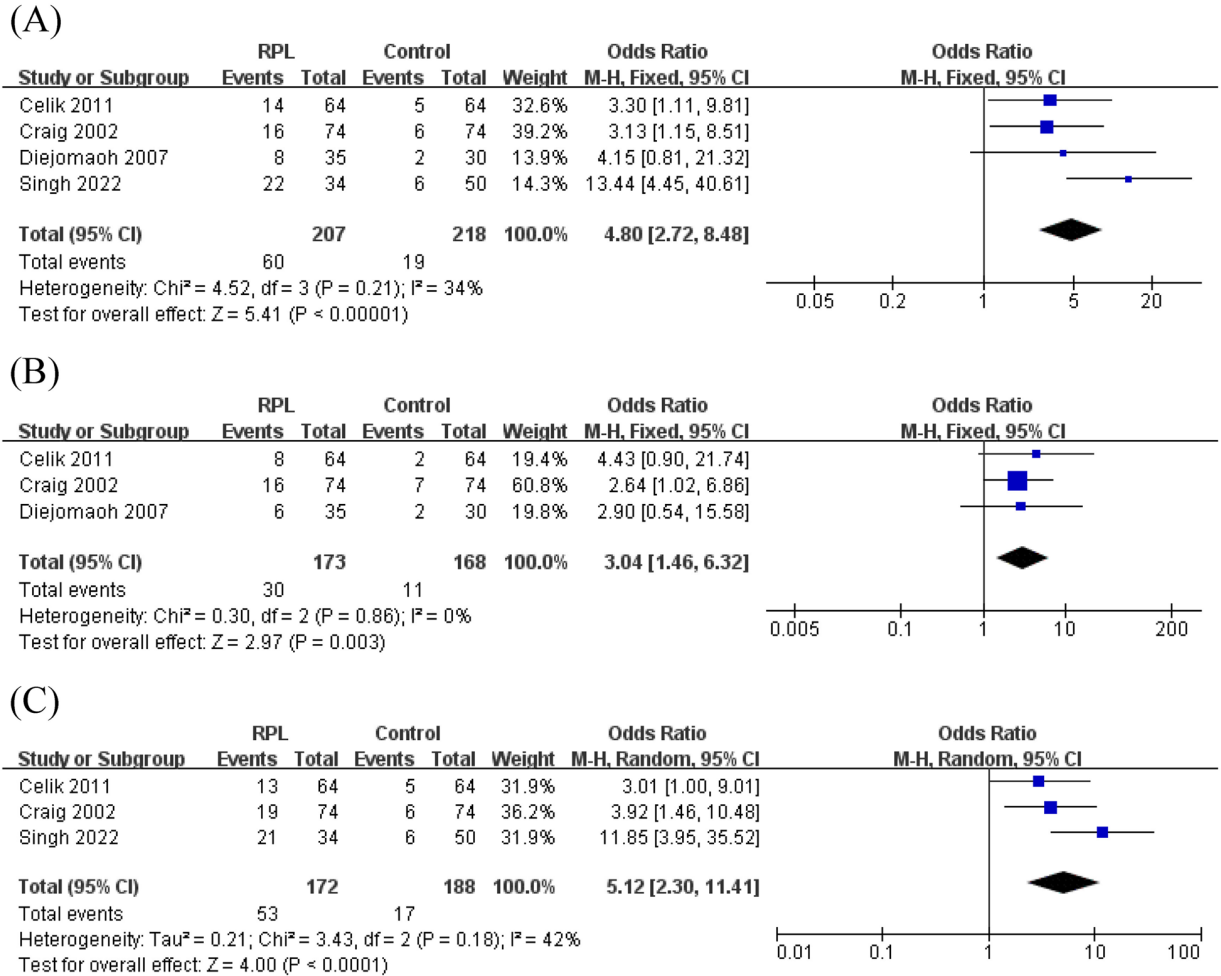
Meta-analysis of IR between the recurrent pregnancy loss and control groups. **A**) HOMA-IR > 4.5, **B**) GI ratio < 4.5, **C**) FIN > 20

The meta-analysis revealed a significantly higher rate of IR in RPL patients defined by abnormal HOMA-IR, abnormal GI ratio and abnormal FIN compared to healthy controls (Fig. 6). The funnel plots showed no obvious asymmetry indicative of a lack of publication bias (Supplementary Figs. 5, 6, 7). The Egger’s line regression test indicated no publication bias for IR when defined by abnormal HOMA-IR (t = -0.02, *P* = 0.986), abnormal GI ratio (t = 0.90, *P* = 0.533) and abnormal FIN (t = 0.50, *P* = 0.705). Subgroup analysis to assess the association between IR status and RPL was not performed due to the limited number of studies included.

## Discussion

The data from this meta-analysis suggested that glucose metabolism was associated with RPL. RPL women had higher FBG, FIN, and HOMA-IR, and a lower GI ratio relative to healthy controls. Additionally, RPL women were observed to have a higher rate of IR status.

First, homocysteine (HCY) and folate have been hypothesized to influence RPL in recent years. In animal and human studies, it has been shown that IR is associated with HCY [41]. One meta-analysis found that high HCY levels and low folate levels were significantly associated with RPL risk [42]. HCY is thought to contribute to blood hypercoagulability [43]. Further, HCY has been shown to inhibit proliferation and promote inflammatory responses in endothelial cells, impair endothelial function, and cause vascular injury [44]. Nelen et al*.* found that elevated maternal HCY is associated with defective chorionic villous vascularization [45]. Additionally, Han et al*.* reported that the exposure of mouse embryos to HCY induced cardiac defects [46]. The authors concluded that HCY may, therefore, be toxic to the embryo. Together, these data indicate that the association between IR and RPL might partly be explained by HCY.

Second, the placenta serves as the main interface between the mother and the fetus and it is understood that peripheral IR status is changed during pregnancy [47]. This increase results in a higher concentration of circulating maternal lipids and amino acids, thus providing glucose and additional nutrients to the fetus via placental transfer and contributing to fetal growth. In obese women during early pregnancy, the human placenta is responsive to the high concentration of maternal insulin. In these cases, this responsiveness is attributed to altered gene expression due to modulated mitochondrial steroid hormone production and energy metabolism [48]. In such pregnancies, IR status may influence placental dysfunction and cause pregnancy loss; however, this needs to be confirmed.

Third, efficient vessel functioning is vital during normal pregnancy. During early human pregnancy, extravillous trophoblast cells from the placenta invade the uterine decidual spiral arterioles and mediate the vessel remodeling to ensure that low pressure, high blood flow can be supplied to the placenta [49]. The development of new blood vessels is also essential to promote ovarian folliculogenesis and functional corpus luteum formation [50]. Insulin is a vasoactive hormone, and evidence suggests that arterial endothelial dysfunction and IR usually coexist [51]. Together, these lines of evidence suggest that IR might disrupt vessel function during pregnancy and lead to pregnancy loss.

Furthermore, IR may impair the procedure from oocyte development to embryo development. IR is associated with decreased percentage of mature eggs and poor embryo quality in [52, 53]. IR may also contributes to oxidative stress and disrupts mitochondrial function in mouse oocytes [54]. High-fat diet- induced IR mouse models had deteriorated uterine receptivity and decreased implantation sites and fetal numbers [55].

Previous study has provided evidence supporting the association between IR and spontaneous pregnancy loss. Tian et al*.* suggested that IR was an independent risk factor for spontaneous abortion in women undergoing assisted reproduction [13]. From our review of the literature, more studies investigated the association between IR and miscarriage in women with PCOS. For example, a recent systematic review and meta-analysis revealed that IR is a risk factor for spontaneous abortion in PCOS patients who underwent assisted reproductive technology [16]. In agreement with these lines of evidence, the results of our study found that IR was associated with women with RPL.

Meanwhile, subgroup analyses showed that geographic region, whether the patients were matched for confounders, and whether the exclusion of known factors might have a significant influence on the association between IR and RPL. The differences observed for the geographic region may reflect ethnic heterogeneity. Matched confounders might have associations with IR and, therefore, influence the subgroup analysis. Additionally, our results indicate that the relationship between IR and RPL was stronger when excluding known factors of RPL, suggesting the possible role of IR in unexplained RPL.

Several factors might influence our results. First, not all confounders related to IR were fully adjusted for in the included studies. For example, the BMIs of all the women were not similar between the RPL and control women. Furthermore, additional confounders that may affect a person’s IR parameters, such as smoking [56] and lifestyle [57] were not evaluated in most studies.

In the current review, RPL women were found to be associated with IR. The screening and prevention of IR may provide health benefits for women with RPL. Al-Biate et al*.* reported that metformin therapy in pregnant women with PCOS was associated with a significant reduction in the rate of early pregnancy loss [58]. Furthermore, in a case report, metformin was shown to be effective in RPL women with IR and PCOS [59]. However, more prospective research is needed to assess if interventions to treat IR can bring long-term benefits to pregnancy outcomes for RPL women.

The search strategy and systematic methods, including quality assessment, publication bias assessment, subgroup analyses and sensitivity analyses are among the strengths of this study. Our study has several limitations. First, the sample size for some indices was relatively small. Most studies were case–control studies, and we are unable to fully access the causality between IR and RPL. Furthermore, the diagnostic criteria for RPL were heterogeneous between studies. Additionally, the quality of several of the studies was not enough. Lastly, some covariates that may affect IR status, such as smoking, lifestyle, were not evaluated.

In conclusion, women with RPL were associated with IR and impaired FBG, FIN, HOMA-IR, and GI ratios. Our study provides improved insight into the understanding of the pathophysiology of women suffering from RPL. The early screening and management of IR may help to improve the pregnancy outcomes of women with RPL and future studies are warranted to further explore the underlying mechanism between IR and RPL.

## Supplementary Information

**Additional file 1: Supplementary table 1.** PRISMA checklist.

**Additional file 2: Supplementary Table 2.** Search strategy.

**Additional file 3: Supplementary Figure 1.** Funnel plot in the meta-analysis on the association of FBG between RPL and control group. **Supplementary Figure 2.** Funnel plot in the meta-analysis on the association of FIN between RPL and control group. **Supplementary Figure 3.** Funnel plot in the meta-analysis on the association of HOMA-IR between RPL and control group. **Supplementary Figure 4.** Funnel plot in the meta-analysis on the association of GI ratio between RPL and control group. **Supplementary Figure 5.** Funnel plot in the meta-analysis on the association of IR by abnormal HOMA-IR between RPL and control group. **Supplementary Figure 6.** Funnel plot in the meta-analysis on the association of IR by abnormal GI ratio between RPL and control group. **Supplementary Figure 7.** Funnel plot in the meta-analysis on the association of IR by abnormal FIN between RPL and control group.

## Data Availability

The datasets used and/or analysed during the current study are available from the corresponding author on reasonable request.

## Abbreviations

RPL: Recurrent pregnancy loss
IR: Insulin resistance
PCOS: Polycystic ovary syndrome
PRISMA: Preferred Reporting Items for Systematic Reviews and Meta-analyses
FBG: Blood glucose
FIN: Fasting insulin
HOMA-IR: Homeostasis model assessment for insulin resistance
GI ratio: Glucose to insulin ratio
NOS: Newcastle–Ottawa scale
MD: Mean difference
OR: Odds ratio
CI: Confidence interval
HCY: Homocysteine
