# Supplementary material for: Insulin resistance in women with recurrent miscarriage: a systematic review and meta-analysis
Source: BMC Pregnancy Childbirth. 2022 Dec 8;22:916. doi: 10.1186/s12884-022-05256-z (PMC9733104; doi:10.1186/s12884-022-05256-z)
Supplement: Supplementary file 2 — Additional file 2: Supplementary Table 2. Search strategy. [file 12884_2022_5256_MOESM2_ESM.docx]

**Supplementary Table 2 Search strategy**

Pubmed

| #1 | (Abortion, Habitual[Mesh]) OR (Abortion, Spontaneous[Mesh]) OR (habitual abortions[Title/Abstract]) OR (Recurrent Miscarriage[Title/Abstract]) OR (recurrent pregnancy loss[Title/Abstract]) |
| --- | --- |
| #2 | (insulin resistance[Mesh]) OR (glucose[Mesh]) OR (insulin[Mesh]) OR (insulin resistance[Title/Abstract]) OR (insulin[Title/Abstract]) OR (glucose[Title/Abstract]) |
| #3 | (Case-Control Studies[Mesh]) OR (Cohort Studies[Mesh]) OR (control[Title/Abstract]) OR (cohort[Title/Abstract]) |
| A total of | #1 AND #2 AND #3 |

Embase

| #1 | ' spontaneous abortion'/exp OR 'recurrent miscarriage'/exp OR 'habitual abortions':ab,ti OR 'Recurrent Miscarriage':ab,ti OR 'recurrent pregnancy loss':ab,ti |
| --- | --- |
| #2 | 'insulin resistance'/exp OR 'glucose'/exp OR 'insulin'/exp OR 'insulin resistance':ab,ti OR 'insulin':ab,ti OR 'glucose':ab,ti |
| #3 | 'case control study'/exp OR 'cohort analysis'/exp OR 'control':ab,ti OR 'cohort':ab,ti |
| A total of | #1 AND #2 AND #3 |

WOS

| #1 | Topic: (spontaneous abortion) OR Topic:( recurrent miscarriage) OR Topic:( habitual abortions) OR Topic: (Recurrent Miscarriage) OR Topic:( Recurrent pregnancy loss) |
| --- | --- |
| #2 | Topic: (insulin resistance) OR Topic: (insulin) OR Topic: (glucose) |
| #3 | Topic: (Control) OR Topic: (Cohort) |
| A total of | #1 AND #2 AND #3 |
