# Supplementary material for: Insulin resistance in women with recurrent miscarriage: a systematic review and meta-analysis
Source: BMC Pregnancy Childbirth. 2022 Dec 8;22:916. doi: 10.1186/s12884-022-05256-z (PMC9733104; doi:10.1186/s12884-022-05256-z)
Supplement: Supplementary file 3 — Additional file 3: Supplementary Figure 1. Funnel plot in the meta-analysis on the association of FBG between RPL and control group. Supplementary Figure 2. Funnel plot in the meta-analysis on the association of FIN between RPL and control group. Supplementary Figure 3. Funnel plot in the meta-analysis on the association of HOMA-IR between RPL and control group. Supplementary Figure 4. Funnel plot in the meta-analysis on the association of GI ratio between RPL and control group. Supplementary Figure 5. Funnel plot in the meta-analysis on the association of IR by abnormal HOMA-IR between RPL and control group. Supplementary Figure 6. Funnel plot in the meta-analysis on the association of IR by abnormal GI ratio between RPL and control group. Supplementary Figure 7. Funnel plot in the meta-analysis on the association of IR by abnormal FIN between RPL and control group. [file 12884_2022_5256_MOESM3_ESM.docx]

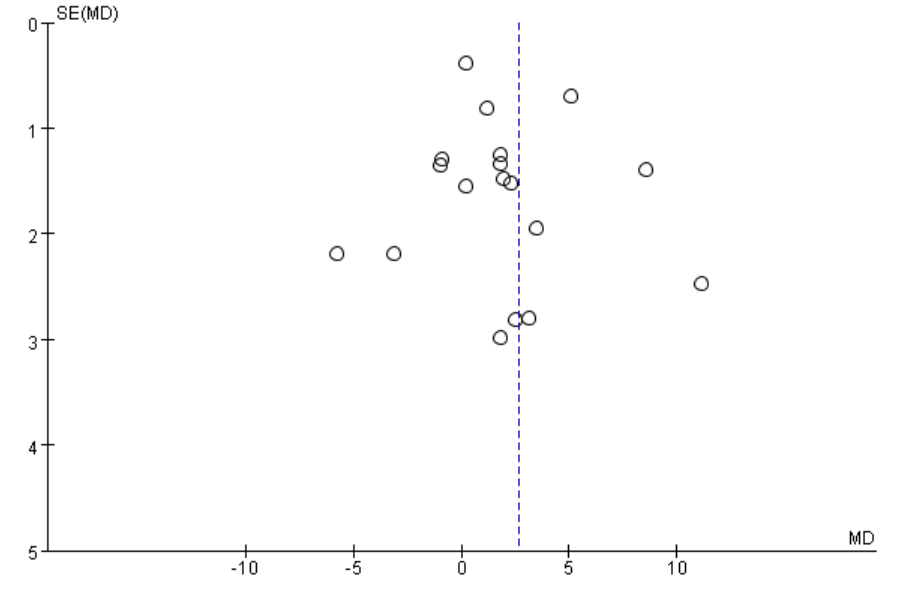


**Supplementary Figure 1** Funnel plot in the meta-analysis on the association of FBG between RPL and control group


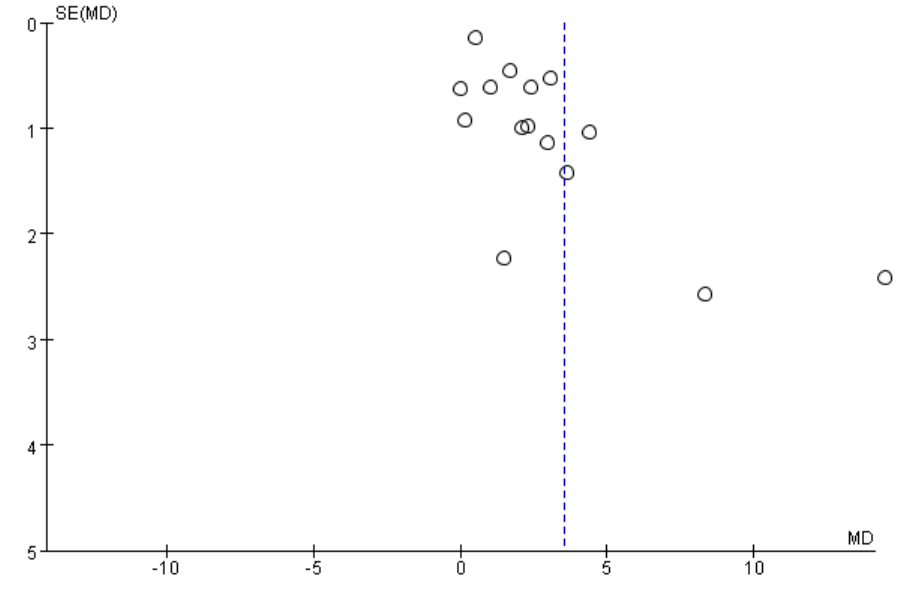


**Supplementary Figure 2** Funnel plot in the meta-analysis on the association of FIN between RPL and control group


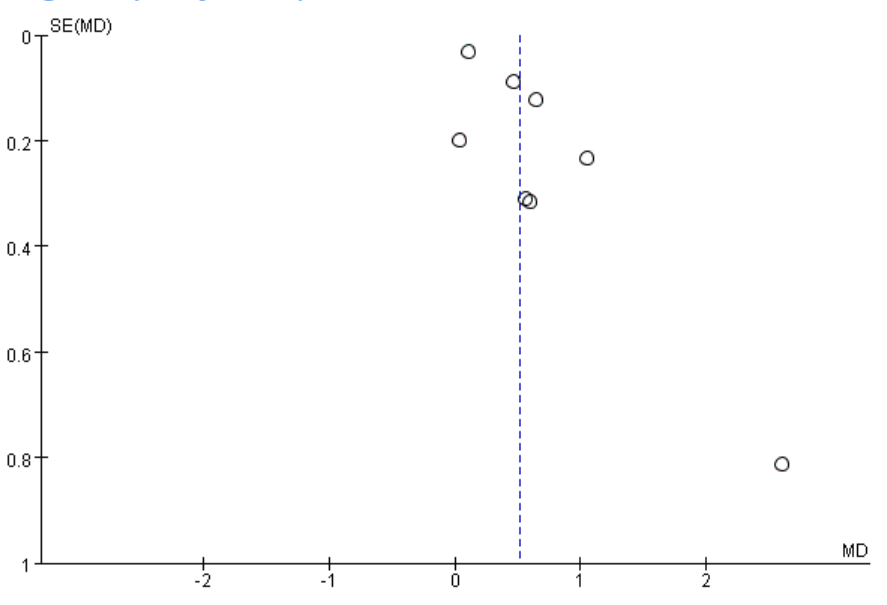


**Supplementary Figure 3** Funnel plot in the meta-analysis on the association of HOMA-IR between RPL and control group


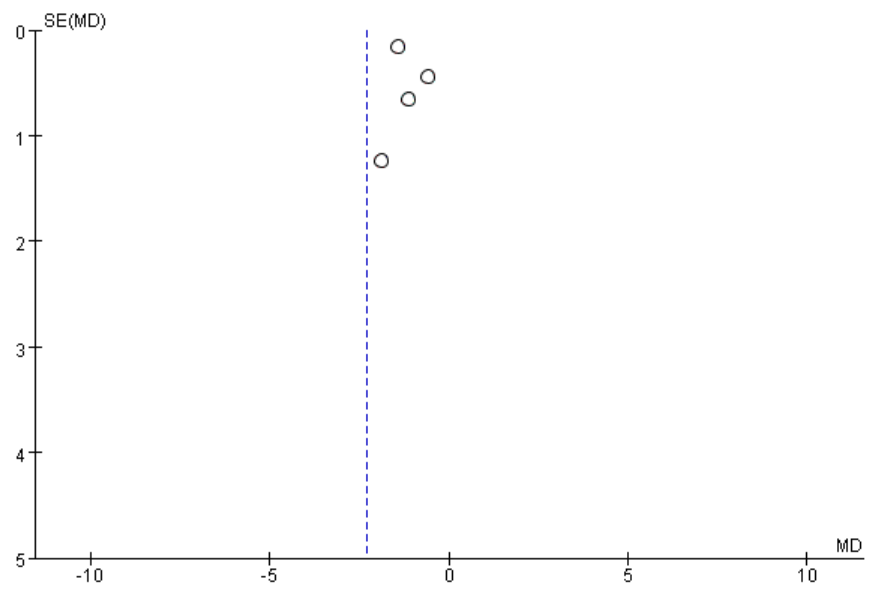


**Supplementary Figure 4** Funnel plot in the meta-analysis on the association of GI ratio between RPL and control group


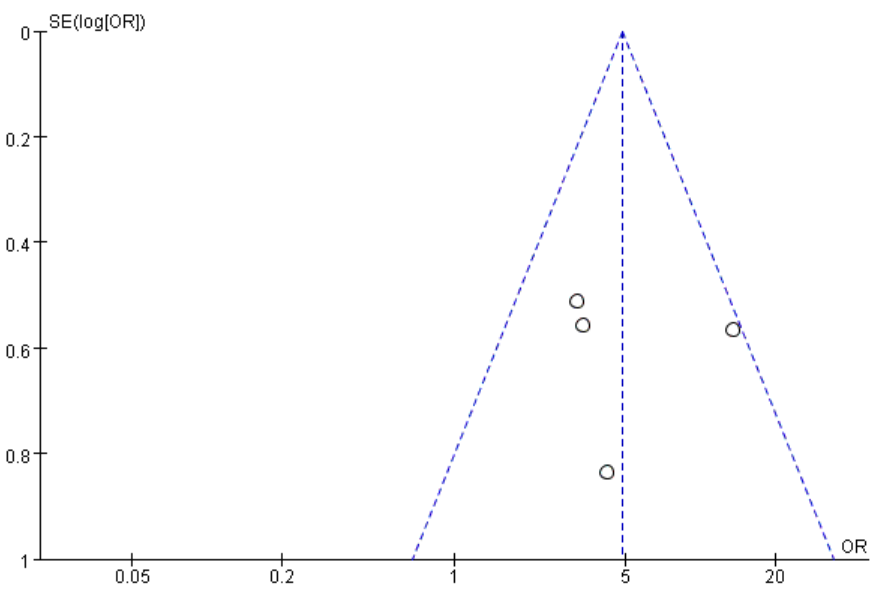


**Supplementary Figure 5** Funnel plot in the meta-analysis on the association of IR by abnormal HOMA-IR between RPL and control group


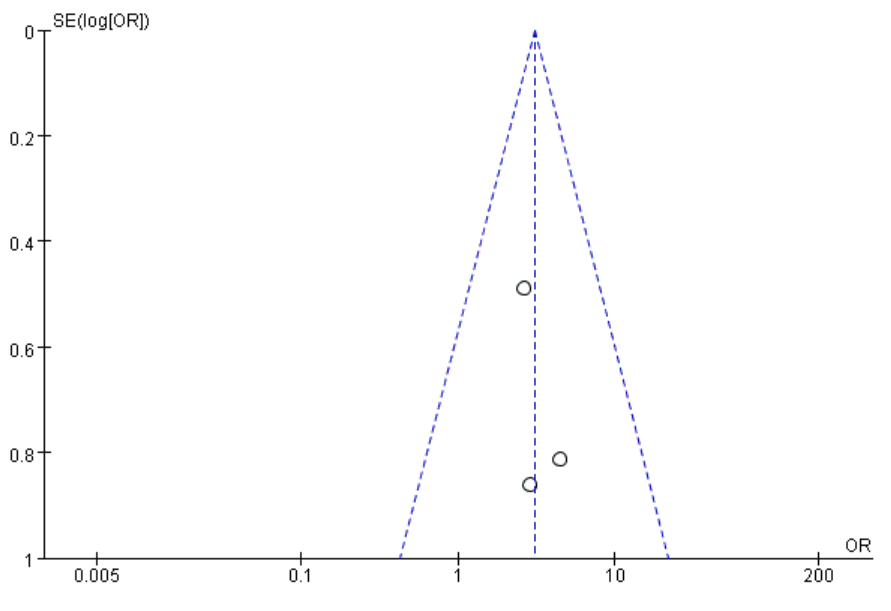


**Supplementary Figure 6** Funnel plot in the meta-analysis on the association of IR by abnormal GI ratio between RPL and control group


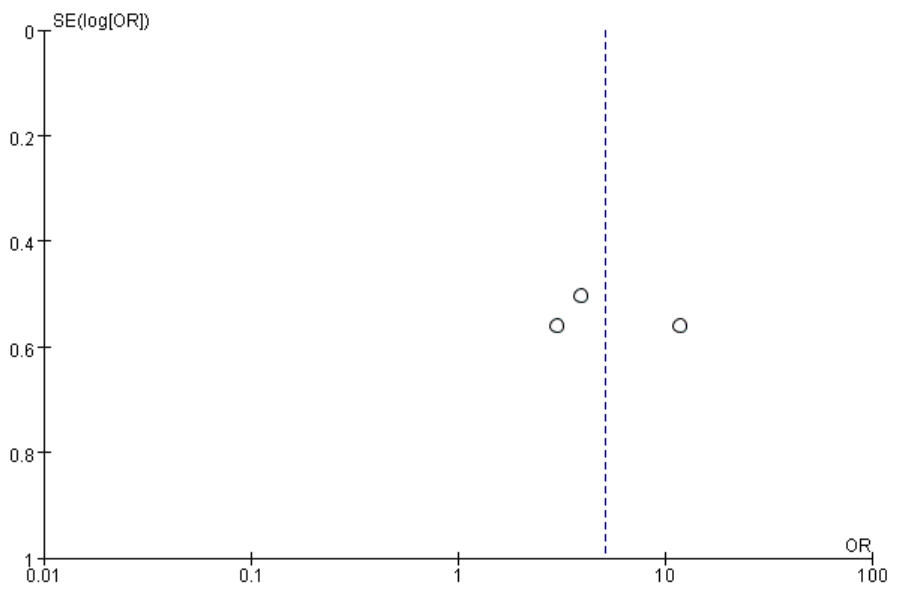


**Supplementary Figure 7** Funnel plot in the meta-analysis on the association of IR by abnormal FIN between RPL and control group
